# The median eyes of trilobites

**DOI:** 10.1038/s41598-023-31089-7

**Published:** 2023-03-08

**Authors:** Brigitte Schoenemann, Euan N. K. Clarkson

**Affiliations:** 1 Department of Biology, Institute of Zoology (Neurobiology, Animal Physiology), University of Cologne, 50674 Cologne, Germany; 2 Grant Institute, School of Geosciences, University of Edinburgh, West Mains Road, Edinburgh, EH9 3JW UK

**Keywords:** Evolution, Neuroscience, Physiology, Zoology

## Abstract

Arthropods typically possess two types of eyes—compound eyes, and the ocellar, so called 'median eyes'. Only trilobites, an important group of arthropods during the Palaeozoic, seem not to possess median eyes. While compound eyes are in focus of many investigations, median eyes are not as well considered. Here we give an overview of the occurence of median eyes in the arthropod realm and their phylogenetic relationship to other ocellar eye-systems among invertebrates. We discuss median eyes as represented in the fossil record e.g. in arthropods of the Cambrian fauna, and document median eyes in trilobites the first time. We make clear that ocellar systems, homologue to median eyes and possibly their predecessors are the primordial visual system, and that the compound eyes evolved later. Furthermore, the original number of median eyes is two, as retained in chelicerates. Four, probably the consequence of a gene-dublication, can be found for example in basal crustaceans, three is a derived number by fusion of the central median eyes and characterises Mandibulata. Median eyes are present in larval trilobites, but lying below a probably thin, translucent cuticle, as described here, which explains why they have hitherto escaped detection. So this article gives a review about the complexity of representation and evolution of median eyes among arthropods, and fills the gap of missing median eyes in trilobites. Thus now the number of median eyes represented in an arthropod is an important tool to find its position in the phylogenetic tree.

## The compound eyes of trilobites

**Figure 1:**
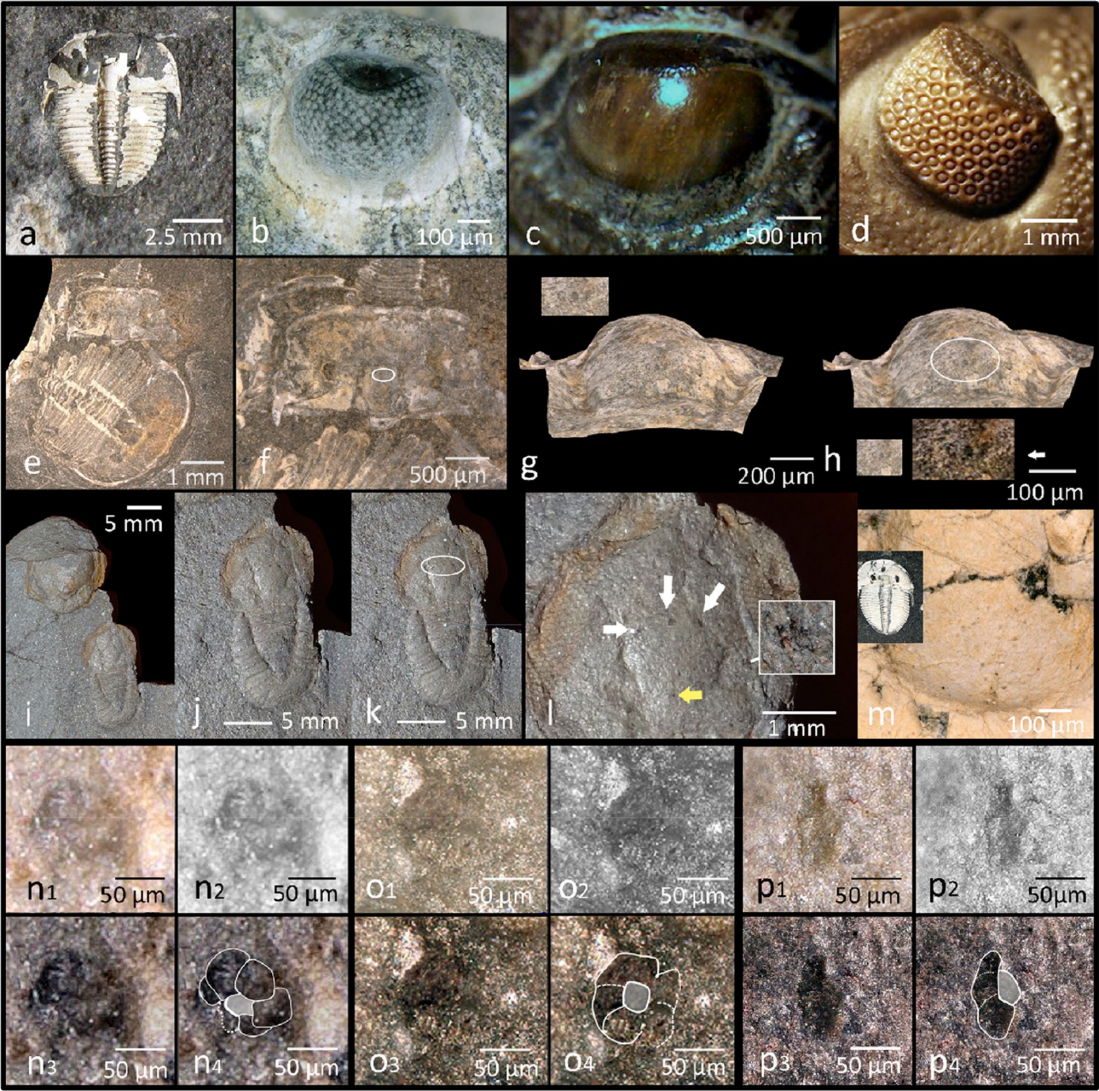
Compound and median eyes of trilobites. (**a**) *Aulacopleura koninckii* (Barrande, 1846), Silurian, Czech Republik, partly with shell preserved. (**b**) Holochroal compound eyes of (**a**). (**c**) Compound eye of *Gerastos cuvieri* (Steininger, 1831), Middle Devonian, Germany. (**d**) Schizochroal eye of *Eldredgeops (Phacops) rana crassituberculata* Stumm, 1953, Middle Devonian, USA. (**e**) *A. koninckii,* two larval stages.** f**) Upper specimen with location of the median eyes indicated. (**g**) Median eyes. (**h**, **g**) enlarged). (**i**) *Cyclopyge sibilla* Šnajdr, 1982, Ordovician, Morocco; 2 specimens, the lower larval. (**j**) Relevant specimen of (**i**). (**k**, **j**) Position of the median eyes indicated. (**l**) Median eyes in (**j**, **k**) indicated by white arrows. Yellow arrow: dorsal organ, comp. (**h**, **i**). Insert: dark structure in the fossil, which is not a median eye.** m**) Fully shelled glabella of *A. koninckii* (insert), no median eyes visible. (**n**_**1–4**_) Left median eye of *C. sibilla* (**l**), different contrasts. (**o**_**1–4**_) Median median eye of *C. sibilla* (**l**), different contrasts. (**p**_**1–4**_) right median eye of *C. sibilla* (**l**), different contrasts.

Compound eyes are typical eyes of euarthropods. Trilobites are extinct arthropods that dominated the faunas of the oceans of the Palaeozoic, and from the very beginning of their appearance in the fossil record they were equipped with very elaborate compound eyes. During 252 million years of successful existence they developed, especially during the Great Ordovician Biodiversification Event (GOBE) different adaptations of these eyes to various habitats and most different predatory pressures. Among trilobites two main kinds of compound eyes have been distinguished. The oldest, most common and longest lasting type is the so-called holochroal eye (Fig. 1a–c). These were first described by Lindström^1^, who distinguished two forms of holochroal eyes—those, in which sometimes up to several thousand lenses, perceptible from outside as facets, in some cases can be recognized, and such, where all facets in common are covered by a smooth cornea. The second main type of compound eye in trilobites is the schizochroal eye (Fig. 1d), present only in the suborder Phacopina. Here the lenses are larger, less numerous than in the holochroal eye, not as densely packed, and often half a lens diameter apart^1–3^.

**Figure 2:**
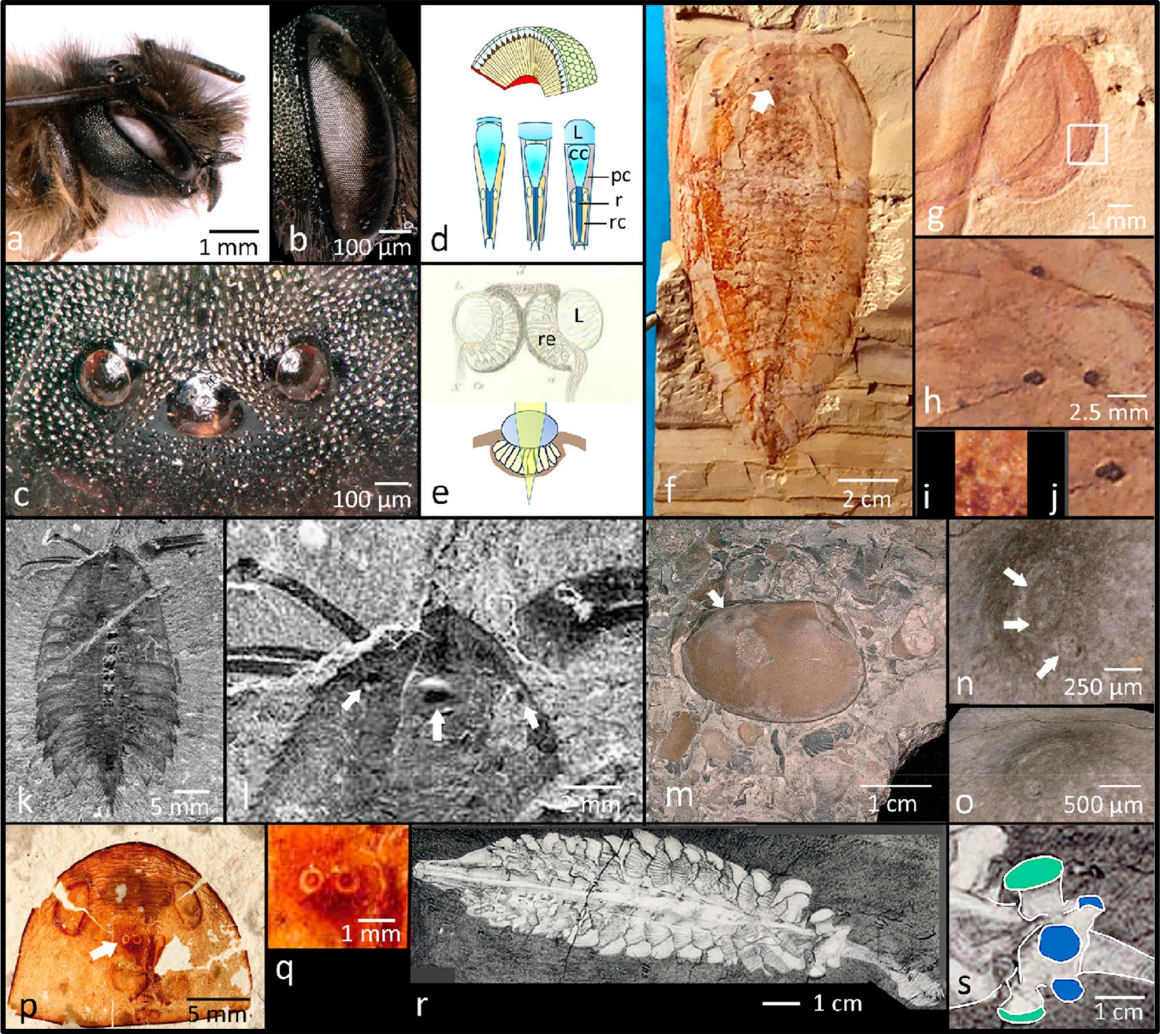
Compound and median eyes. *Osmia cornuta* (Laterille, 1805). (**b**) Compound eye of (**a**). (**c**) Median eyes of (**a**). (**d**) Schematic drawing of a compound eye, ommatidia of non-malacostracan and malacostracan crustacean and of an insect (after^117^). (**e**) Schematic drawings of an ocellus. Upper part prob. *Cypris pubera* Müller 1776 (Ostracoda)^118^, Plate 1.3. f) *Cinderella eucalla* Chen et al*.*, 1996. (**g**) Lower Cambrian, China, right compound eye of (**e**). (**h**) Four median eyes (white arrow in **f**). (**i**) Hexagonal facet (rectangle in **g**), diameter ~ 80 µm. (**j**) Cup-like ocellus, diameter ~ 80 µm. (**k**) *Leanchoilia superlata* Walcott, 1912^103^, Fig. 2B9, Middle Cambrian, Canada, Burgess Shale. (**l**) Median eyes of (**k**), note the fused ones in the middle. Arrows: median eyes. (**m**) *Hermannina* sp. (Ostracoda), Silurian, Sweden. Arrow: Eye. (**n**) Section of (**m**), arrows indicate three median ocelli, here functioning as main eyes. (**o**) three dimensional aspect of the system. (**p**) Head of *Eysyslopterus patteni* (Størmer, 1934), Eurypterida, Silurian, Estonia^47^, plate 1, arrow indicates (**q**) two median eyes. (**r**) *Opabinia regalis* Walcott, 1912, Middle Cambrian, Burgess Shale, Canada^99^. (**s**) Three median eyes (blue) and two lateral eyes (green) of (**r**). *cc* crystalline cone (part of the dioptric apparatus), *L* Lens, *pc* screening pigment cells, *r* rhabdom (light perceiving structure), *rc* receptor cells, *re* retina.

During the last years it has been possible to clarify the functional structure of both kinds of eye. The holochroal eye of *Schmidtiellus reetae* Bergström, 1973 from the lower Atdabanian, early Lower Cambrian of Estonia, shows the typical arrangement of structural elements, as characteristic of the apposition eyes of living diurnal crustaceans and insects^4^. The apposition compound eye consists of typically numerous identical units, so-called ommatidia. Here c 8 receptor cells are arranged around a central light guiding structure, the rhabdom. The light is focused onto this rhabdom by a lens, in trilobites of primary calcite through a clear, cellular crystalline cone (Fig. 2d)^3–5^. Within the rhabdom containing the visual pigments the light signal is transformed to an electrical signal, to be processed by the central nervous system. Over the entire compound eye a mosaic-like image emerges, comparable to a picture formed by a computer screen, with each ommatidium contributing one 'pixel'. This system is therefore comparable to the apposition eyes of today's diurnal insects and crustaceans. Schizochroal eyes, enigmatic for a long time, are hyper-compound-eyes. In these, about seven, sometimes more small ommatidia, each of them equipped with receptor cells, a crystalline cone and a small lens, have gathered under each of the big lenses. Thus, each of these thick lenses seen from outside covers a small compound eye of its own^6^. The functional adaptation of this system still is not understood completely.

## Two types of eyes in living arthropods

Even if the compound eyes are plesiomorphic and the most conspicuous visual organs of arthropods, these are not their only light perceiving organs. Arthropods typically possess two types of eyes—compound eyes (Fig. 2a,b), and the ocellar, so called 'median eyes' (Fig. 2c). Perhaps surprisingly, trilobites do not seem to have any such. In some cases there are the so-called frontal organs. Frontal organs were originally photoreceptor-organs lying aside of the median eyes, but cannot be considered as 'eyes' in the narrow sense^7^, because they do not possess screening pigments, isolating the receptor cells optically^8^, p. 482. Frontal organs are present at least in Crustacea and Collembola, and are innervated by the same inner part of the protocerebrum as are the median eyes. Functionally often they change during ontogeny to endocrine organs, for example such as the head glands of many myriapods (Gabe Organ), or the X-, Y-organ of crustaceans. As a result their relation to median eyes remains unclear^9,10^. There is a very detailed discussion about the complex and highly diverse situation of median eye- and frontal organ systems of recent arthropods in the work of Elofsson^11–13^, and Paulus^10^, a detailed discussion about structure and function of dorsal organs see Supplement 1.

If we follow Gehring´s conception^14^ that as in the Cambrian lobopod *Microdictyon* sp. in the panarthropod ancestor each (proto-)segment was equipped not just with one pair of limbs, but also with a pair of compound eyes^15^, as well as paired internal organs, one may expect that after cephalization, when three protosegments fuse to a head, the cephalon of an advanced arthropod finally should possess at least six eyes, while the 'limbs' change by diversified Hox genes to three pairs of differentiated mouth appendages. Most indicative here is the situation in the primitive brachiopod crustacean *Triops* sp. (Notostraca) which has survived virtually unchanged since the Triassic. In total it possesses 6 eyes (plus 4 frontal organs): 2 compound eyes, and 4 median eyes (plus 2 dorsal frontal organs, 2 ventral frontal organs^16,17^). [The function of *Microdictyon*’s, originally organic, and just secondarily phosphatic plates has been much discussed and is still contoversal^18^. These segmental sclerotic plates, here interpreted as compound eyes, also have been variously discussed as limb attachment points^19,20^ or as protective devices^21^. Meanwhile it seems more or less accepted, however, that the segmental, lateral sclerits of *Microdictyon* sp. are homologue to those of other lobopodians, such as *Onychodictyon* sp. or *Hallucigenia* sp., where the sclerotic plates and spines surely were protective. They are comparable to very similar net-like sclerotic plates of some lower Cambrian palaeoscolecid worms, such as *Cricocosmia* sp. or *Tabelliscolex* sp., living in the ground. Here these plates also have been discussed as defensive organs^22^. Another function may be that the plates stabilize the shape of the hydro-skeleton of these small worm-like organisms mechanically like a Pfeffer´s Cell, against influences of quickly changing osmotic conditions, when hyperosmotic outer conditions may easily let them get flubby.]

## Simple eyes, especially median eyes among arthropods living today

### Phylogenetic retrograde view on median eyes

Median eyes are small cup-eyes (ocelli), floored by a more or less complex retina (Fig. 2e), often covered by a lens. In phylogenetically advanced arthropods such as insects for example, eponomously they lie between the compound eyes. Median eyes are plesiomorphic for panarthropods and not homologous to the larval stemmata of holometabolous insects, which actually are fused compound eyes^24^. The number of median eyes among euarthropods varies, reflecting evolutionary changes. In crustaceans this eye is the larval tripartite, so-called Nauplius eye, which in adults is only preserved in copepods, and in most ostracodes. In the latter they even form the main eyes (Fig. 2m–o). Three median eyes are the most common type^10,25,26^, and it is only the Nauplius eyes of most Phyllopoda that consist of four median eyes^27^. Insects generally show three median eyes (Fig. 2c)^28^. It is only in Collembola that six ocelli are present^9,10^ which are, however, visual organs of different types. Corresponding with crustaceans all Hexapoda show three median eyes, which presumably arose by fusion of the median ocelli of the original four^9,10^. Thus, these three ocelli may be understood as synapomorphies of Crustacea and Hexapoda, i.e., an autapomorphy of Tetraconata/Pancrustacea (sensu Dohle 2001^29^)^30^. In total it seems that the first visual systems that equipped panarthropods were ocelli (see lobopodians), then compound eyes appeared. Ocelli, retained as median eyes, kept on co-existing with compound eyes during the course of evolution.

The function of median eyes is diverse and not completely understood yet. Apart from dragonflies it seems that all median eyes of insects underfocus (the focal plane lies behind the light-perceiving layer of the retina), and although being equipped in some cases with a reflecting tapetum and iris, a field of view of 150° and sometimes as many as 10,000 photoreceptors, such underfocusing, forms a blurred image. There seems to be a more or less general consens that in flying insects the dorsal ocelli are horizon detectors supporting flight equilibrium^31^, p. 127. For marine organisms, however, this interpretation probably makes no sense. A good example which gives a conception of how these organs may have functioned in Palaeozoic arthropods is given by the well investigated xiphosuran *Limulus* sp*.* In the xiphosuran the situation is complex. It has one pair of median eyes^32–34^, and a fused pair of so-called endoparietal eyes underneath, which are considered to be rudimentary median eyes^35,36^. In larval stages there is a third pair of ocellar eyes close to the brain, merging later with the frontal organ. As a result there probably were 4 median eyes originally, and they all are innervated by the same center within the central body of the brain^37^. There are two ventral photoreceptors in early instars also, which, however later change to olfactory organs^38–40^.

The receptors of the median eyes are sensitive to visible, but also to ultra-violet radiation. The sensitivity of the lateral and median eyes is controlled by a clock in the anterior part of the brain, while signals from the median eyes enhance the degree of adaptation to darkness in the lateral eyes according to the amount of UV-radiation reflected by the moon at night^41^. Because UV-radiation attenuates sharply with water depth, it well may be that this function is used to control the residence of horseshoe crabs within a specific range of depth in the sea^42^. Furthermore, horseshoe crabs use their compound eyes to trace up mates^43^ during the night, thus the median ocelli may enhance the spawning process (for an overview see Batelle^44^). The function as a kind of 'setter' for the lateral eyes during the night can be imagined as a useful support for vision of ancient marine animals in the same way.

In Pancrustacea (Crustacea and Hexapoda) the cephalon is built by three segments, as is reflected by the tripartite brain which consists of the proto-, deutero- and tritocerebrum. Regardless, whether the head arises by fusion of three thoracic segments or whether it evolved from a duplicated single-segment head, not homologous with any thoracic segments^45^, there remains the problem that frontal organs and median eyes are innervated by neuropiles separate from those of the compound eyes. All centers lie within the protocerebrum and are not positioned serially according to the tagmata. Evolution, however, generated a great plasticity in forming brains, and it probably is of great functional advantage if the spatial distance between all these visual centers is as short as possible. Lev, Chipman and colleagues give a comprehensive review of the current discussion on cephalization in arthropods^45,46^.

Myriapodes do not possess median eyes at all, which, in the context of their generally reduced eye system, may be seen as an adaptation to their habitat (darkness, litter).

Within the Chelicerata the fossil eurypterids clearly show just 2 median eyes^47,48^ as do all extant chelicerates^49,50^. The conservative Pycnogonida are equipped four median eyes^35^, and the fact that the four ocelli in pycnogonid larvae are innervated by a single one, but bifurcated nerve^51^ may indicate the evolutionary pathway—it is likely that the chelicerates retained the original number of 2. In some spiders (Salticidae), the dorsal median eyes become the main eyes with a complex optic and retinal system^52–54^, while the compound eyes decay and single ommatidia fuse and build own camera eyes ('side eyes') of different numbers^10^.

*Onychophorans* are ecdysozoans (invertebrates moulting a chitinous exoskeleton), and are generally considered as closely related to arthropods and tardigrades, together forming the taxon Panarthropoda. As their Lower Cambrian relatives the lobopodians, onychophorans possess one pair of small (0.2–0.3 gm) camera eyes, with clearly a distinct lens^23,30,53,55–57^. The ocellar eyes lie at the dorsal base of the 'antennae' (the latter are probably not homologous with the antennae of arthropods, or chelicerae of the Chelicerata, but may find an equivalent homologue on the frontal-filaments of some crustaceans, such as Remipedia, Cirripedia or Branchiopoda^58^, p. 454.) These eyes consist of pigment cups^30,55,56^. The ocelli are each filled with a gelatinous lens, and the entire structure is covered by a translucent epidermis. In onychophorans the cephalization has not proceeded as far as it has in insects for example. The brain does not consist of three neuromeres, only two of them are present^59^. By contrast with compound eyes these ocelli develop from an ectodermal groove corresponding to the median eyes of euarthropods. They are associated with the central part of the brain rather than the lateral region where compound eyes innervated^30^. This central part is very similar to the arcuate body of chelicerates (sensu Strausfeld^60,61^), differing from the central body of pancrustaceans by their internal neuroarchitecture, their consistent cell-types, and the position of the neuropils within the brain. Both centers, however, are connected to the median eyes of crustaceans and insects, rsp. chelicerates, and at most indirectly with the compound eyes^60–62^. Immunohistochemical experiments have recently confirmed the validity of the hypothesis that onychophoran eyes are homologous to the arthropod median ocelli^63^.

Functionally, the eyes of velvet worms underfocus, (as do most lens-equipped ocelli with a small retina directly below the lens). This means that the focal plane lies behind the light-perceiving layer of the retina, and thus the received image is blurred. It makes a low-pass filter, where just the rough patterns of the environment can be recognized, while details cannot be resolved. This may be a good adaptation for a poorly differentiated brain, such as is possessed by many of these small invertebrates^23,53^.

Probably close relatives of the velvet worms (Onychophora) and arthropods are the tardigrades, with which they form the taxon Panarthropoda. Many Eutardigrada and some Arthrotardigrada, namely the Echiniscidae, possess inverse pigment-cup ocelli, which are located in the outer lobe of the brain, and comprise one or a few rhabdomeric (microvillous) and ciliary sensory cells^64^. Erlanger reports that *Mac*r*obiotus macronyx* Dujardin, 1851 (Eutardigrada, Parachela) possesses a 2 µm in diameter pigmented ocellus which even has a gelatinous hemispherical lens^65^, documented by Kristensen^66^ also.

The tardigrades possess a brain with distinctly paired regions (lobes), most authorities agree with the existence of a pair of outer, and a pair of inner lobes. Because the brain combines the connectives of 3 ½ segments, the whole brain may be considered as homologous to the protocerebrum of arthropods^64^, p. 466. The elongation of the prominent outer lobes extends in the caudal region, innervating a sensory area (temporalia) and the ocellar eyes^67,68^, p. 385.

### Annelids relation to the rest

Lastly, annelids are equipped with a chitinous outer membrane, but they do not moult. Annelids possess three types of photoreceptors—rhabdomeric, ciliary photoreceptors and phaosomous. The rhabdomeric type occurs mostly together with supportive pigment cells, while both of the other types do not^69^. Annelid eyes range from diminutive structures of one or two receptor cells up to large camera eyes with a vitreous body, elaborate lenses and multicellular retinas^69–71^. Mostly these eyes sit close to the cerebrum, they can be everse or inverse and all are ectodermal. Most sophisticated eye systems occur among sabellid and serpulid polychaetes^72–74^ and the pelagic predatory polychaetes of the genus *Vanadis*^70,75,76^. The innervation of these eyes occurs through the middle part of the simple brain^77^, p. 364.

Many forms of annelids possess light-perceiving organs all over the body. Typical are so-called phaosomes^77^, p. 411. In its head-region *Erpobella octooculata* (Linnaeus, 1758), (Hirudinida) for example possesses eight pairs of eyes close to the brain, each consisting of a pigment cup, open to the front, filled with 24–35 receptor cells. These receptor cells have an inverted membrane with a rhabdomeric rim protruding into a gel-like cavity (phaosome)^78^. The nerve leaves the cup on the opening side (everse). The nerves are connected directly with the middle region of the oesophageal ganglion, as are the nerves of the antennae (e.g. *Saccocirrus* sp., Saccoricidae)^79^. The most complicated eyes in annelids are shown by fan worms, sabellid and serpulid polychaetes. On their feeding appendages they build compound-eye-like arrays of sensory organs, sometimes with sophisticated optics^72–74^.

Because of their segmentation, formerly grouped with the onychophorans as articulates^80^, the annelids are now regarded as Lophotrochzoans based on the formation of a Trochophora-larva and molecular-biological investigations^81^. Annelids show a concentration of light receptors in the head area, but the further development of more complex light-sensing organs is convergent to the panarthopoda.

Thus in total one can observe a consistent phylogenetic lineage of the ocellar median eyes from the ocelli of onychophorans to the median eyes of euarthropods. Although the segmental composition and evolutionary development of the arthropod brain is complex and not yet understood completely^82^, the innervation of the ocelli is provided by corresponding parts of the brains. It is always from the anterior part of the protocerebrum, respectively by corresponding homologous parts of the central ganglia, while compound eyes always are innervated by laterally and posteriorly positioned nerves. A molecular characterization of the embryonic origin of median and compound eyes in the common house spider (*Parasteatoda tepidariorum* (Koch, 1841)) shows that within the eye-antennal domain both visual organs are determined in non-overlapping domains^50^. The primordia of both visual organs are formed in non-neurogenic ectoderm at different places, developing largely independently. Those of the median eyes start in an anterior median position in the developing head while the lateral eyes start from a lateral position^50^. This principle also is well known from the fruit-fly *Drosophia melamogaster* Meigen, 1830^83,84^, indicating that for median and lateral compound eyes a unique core determination network may have been in place already in the last common ancestor of Chelicerata and Pancrustacea/Tetraconata^50^. Trilobites today are seen as a separate branch in the phylogenetic tree between Chelicertata and Mandibulata^85^. There are strong arguments, however, to assign them to tetraconats, because they possess a crystalline cone^4,85–89^, and consequently, there should have been median eyes in trilobites, too.

Following our retrograde view through the representation of ocellar median eyes and their homologuous predecessors through phylogeny based on representatives of today-living organisms, it seems to be probable that the median eyes had been present even earlier than trilobites. So—where are the median eyes of trilobites, and are they present anywhere in the fossil record?

### Fossil median eyes

The high diversity of elaborate compound eyes in the fossil record, especially of trilobites, but also of radiodonts, megacheirans and other arthropods of uncertain assignment, such as *Isoxys,* has given rise to numerous reports about their structure and function^90–98^, and many aspects of their structure and function are well understood. Median eyes, however, as mentioned, a second probably plesiomorphic visual organ of arthropods, have received less attention. Here may demonstrate some examples of their early existence, sometimes clarifying their uncertain documentation.

Recent descriptions of the Cambrian megacheirans *Leanchoilia* sp. and *Alalcomenaeus* sp. have proved highly controversial.

Tanaka and colleagues^94^ describe 4 compound eyes for *Alalcomaneus* sp. and interpret the multiplicity of eyes as typifying chelicerates, but no median eyes were described as such. [More recent analyses show that leanchoilids and alacomeneuids are not chelicerates but megacheirans (in the tree they are before the split between chelicerates and mandibulates)]^85^. The authors apply this concept also to *Leancholilia superlata* (Walcott, 1912) and *Leanchoilia persephone* Simonetta, 1970, seemingly based on the works of Garcia-Bellido and Collins^90^, and Haug and colleagues^98^ alone. Specimens of *L. superlata*, then newly described and illustrated by Haug^98^, show the pedunculate eyes clearly in the lateral view (there Fig. 2B). The authors describe them as lateral eyes, each with short stalks arising from the antero-ventral region of the head (there Fig. 3D,F–H), and consisting of two lobes. Fig. 3G^98^ clearly indicates 4 median eyes, identical to those as described by Garcia Bellido and Collins^90^, but here only referred to as 'eyes' with no closer discrimination. Garcia-Bellido and Collins give a comprehensive review of the history of the discussion of *Leanchoilia*’s eyes. Walcott's original account reported on “a large pedunculated eye comparable to that of *Opabinia regalis* Walcott, 1912^99^, p. 171. Raymond reported on large, reniform depressions 'likely the remains of very large, sessile compound eyes' although 'no lenses are visible'^100^, p. 213. These were very probably the ocellar median eyes. None of the later descriptions showed any eyes at all^101–103^, and consequently the famous reconstruction of Marianne Collins in Gould^104^ showed a blind *Leanchoilia *sp. Garcia-Bellido and Collins^90^ point out, that the four median eyes were difficult to find under the microscope, and only showed up under bright sun light, or transverse light, because these eyes lie near the front of the ventral underside of the head shield. The authors suggest that this position led to the fact that these eyes had not been previously recorded. Because these eyes, the outer pair being larger than the inner, have no facets, the authors interpret them correctly to be median ocelli. This interpretation is in accordance with the accounts of Hou and Bergström^105^, and Schoenemann and Clarkson^4^. The latter described penduculate compound eyes, and four median eyes.

Thus one may conclude that the genus *Leanchoilia* possessed penducuIate compound eyes, in *L. superlata* they may have been even bilobate^98^, and may have possessed four ocellar median eyes. Bilobate, penduculate compound eyes were described for *Alalcomenaeus* sp.^94^ also. There is, however, an excellent figure of *Alalcomenaeus cambricus* Simonetta, 1970 given by Briggs and Collins^106^, Figs. 4 and 5.4, showing a large, club-shaped stalked compound eye, which clearly is not bilobate. In consequence the question of bilobate or 'mono-lobate' penduculate compound eyes in *Leanchoilia* sp. and *Alalcomenaeus* sp. remains somewhat enigmatic.

Clearly, however, *L. superlata* and *L. persephone* from Burgess Shale additionally possess four ocellar median eyes, the outer pair larger than the inner. They are not documented for *Leanchoilia illecebrosa* (Hou, 1987) from the Maotianshan, China, probably because delicacy of the structure and different mode of preservation. *A. cambricus* possesses three median eyes^106^, pointing the way towards the pancrustacea. There is one interesting specimen of *L. superlata*, shown by Butterfield^107^, revealing the fusion of the inner median eyes (Fig. 2k,l), perhaps a transition to the typical number of 3 median eyes as typical for pancrustaceans also^108^.

The mandibulate *Waptia fieldensis* Walcott, 1912 (Burgess Shale)^109^, and the crustacean *Odaria alata* Walcott, 1912 (Burgess Shale) possess three median eyes, comparable with those of Pancrustacea^96^. Most ostracodes (Podocopia) of today possess a single visual apparatus consisting of three median eyes. The ocellar cups are situated near the anterior end of the hinge, just above the base of the antennules. The Silurian *Hermannina* sp., (Leperditiidae, Ostracoda), Lickershamn, Gotland, Silurian possesses a visual apparatus consisting of three median eyes (Fig. 2m–o). Some ostracod groups such as myodocopids, additionally display a pair of stalked compound eyes situated laterally below a translucent cuticle.

As mentioned eurypterids possessed two median eyes, and it is likely that the chelicerates retained the original number of 2.

Even the enigmatic situation in *Opabinia regalis* Walcott, 1912, (Burgess Shale) with its five eyes, now becomes understandable, for they can probably be interpreted as 3 median eyes and 2 lateral compound eyes. Whether the latter are compound or ocellar eyes needs further consideration (Fig. 2r,s). At least, due to their lateral position, they seem to be homogues to the compound eyes of euarthropods.

One of the most clear and meaningful examples in this context is given by the median eyes of *Cindarella eucalla* Chen et al., 1996 (Fig. 2f–j), classified within the stem group of trilobites, as an element of the arachnate diversity^110^. Here we find four median eyes in the middle of the cephalon, which clearly show the typical shape of ocelli, namely a distinct cup-like structure^101^.

### Median eyes in trilobites

Median eyes as such have never been documented in the trilobite literature. The only previous author who reported them was Ruedemann^111^ in 1916, but he did not illustrate them, and there is no distinction between dorsal organs and median eyes in any former reports. Both authors of the present article worked for several decades on trilobites, including their sensory organs; Euan Clarkson had described many of them in great detail, but had never observed median eyes. There are two possible explanations for why this is so. The first is that median eyes, by comparison with the situation of most adult crustaceans, were never present in trilobites. The second is that they may have been overlooked because they are inconspicuous. Median eyes normally are very small, just some tenths of micrometres in size. As compared to compound eyes they have hardly any structure by which they could be distinguished from other dark structures in a petrified fossil, and, in the worst cases, as comparable to *Leanchoilia* sp. or ostracods, they may be hidden under the cuticle. If any were found, one might expect, however, structures regularly arranged in numbers of two, three or four, more or less round or oval. Median eyes in living arthropods contain pigments. Consequently, when median eyes were observed externally, one would expect dark structures which are, among other components, the relicts of melanine or related pigments, stable over a long time period^112,113^, showing in total a cup-like, round or oval shape as the remains of an ocellus. Such structures would be expected to lie anteriorly to the compound eyes.

### New evidence on median eyes in trilobites

#### *Aulacopleura koninckii* (Fig. 1a)

A slightly abraded cephalon of *Aulacopleura koninckii* (Barrande, 1846) shows, at the front of the glabella, three almost identically shaped dark, unconspicious tiny oval spots of equal (~ 30 µm wide, ~ 50 µm long) size (Fig. 1e–h). These three structures are lined up in parallel, slightly fanning out on the underside. All of the three spots are characterised by a smooth, clear outline, and an equal, homogenous dark brownish colour. This clear, regular appearance distinguishes this structure from accidental formations resulting from decay or finally fossilisation, but matches perfectly the characteristics, as explained above, to be expected for median eyes. Even if this is an isolated discovery, it supports the concept that median eyes were originally present in trilobites. The slight abrasion of the cuticle opens a clearer perspective, indicating that the median eyes in trilobites lie, as in *Leanchoilia* sp. or ostracodes, below the cuticle, in the fossil invisible from outside (Fig. 1m). The cuticle in vivo probably was translucent. The median eyes were found in a specimen at an early stage in development, and because they had never been observed before, one may assume that as in crustaceans, it is quite possible that only the early developmental stages of trilobites possessed median eyes—a reason why they have not been detected previously.

#### *Cyclopyge sibilla*

Another possible example is shown in *Cyclopyge sibilla* Šnajdr, 1982 (Fig. 1i–l, n–p). Here we find on the glabella three slightly squeezed, formerly probably cup-shaped dark structures on the glabella, which we interpret as median eyes. These structures by their distinct, and trifold repeated form are very different from other undifferentiated dark spots in the fossil following the surface-irregularities of the fossil (Fig. 1l insert). All of the presumed relicts of median eyes here consist of a group of about six cells with a central element, presumably a lens. So these median eyes of this pelagic trilobite seem to have been more complex than those of the benthic *Aulacopleura* sp., and probably had more distinct functions—perhaps similar to those of *Limulus *sp.

Because the upper part of the specimen is covered by a part of a larger trilobite of the same species, it is reasonable to assume that the median eyes here pertain also to a larval stage.

## Conclusions

In summary one may conclude that median eyes were really present in trilobites. That the described structures of trilobites, and of the other palaeozoic arthropods analysed here, are indeed median eyes is concluded by structural comparison to median eyes of to trilobites related extant arthropods. Their median eyes also consist of small retinal layers or cup-like ocelli, sometimes equipped with a simple lens, and lie in a median position of the cephalon. In trilobites, there were three such eyes, as typical for euarthropods, not four in number, as in some earlier forms. These median eyes consisted of cup-like ocelli, also typical for euarthropods. In *Aulacopleura* sp. they lie at the front of the glabella, oriented anteriorly, in *Cyclopyge sp.*, which swam upside down, they are positioned on top of the glabella, consequently directed downwards. The median eyes of the pelagic trilobite (*Cyclopyge* sp.) seem to be more elaborated than those of the benthic trilobite (*Aulacopleura* sp.), because they seemed to have possessed a lens (Fig. 1n–p). Both median eye-systems were found in earlier instars of trilobites, and not in adult individuals. An occurrence, as comparable to many modern crustaceans, only in larval stages, would explain why the median eyes had been overlooked so far—the adults, historically more fully investigated, probably do not have them. Because we found both systems in slightly abraded specimens, one may assume that, as comparable to *Leanchoilia* sp. and ostracodes the median eyes lay below a translucent cuticle. When fossilised this cuticle becomes opaque, and thus makes the structures below invisible.

It seems evident that median eyes are homologous to the eyes of the ecdysozoan onychophorans^30,63^. Eurypterids, as most other chelicerata show two median eyes^10,47,58^, p. 500, (Fig. 2p,q), and it is likely that the chelicerates retained the original number of 2.

The xiphosuran *Limulus* sp. with its high diversity of eyes (two lateral compound eyes, two median eyes, one endoparietal eye (= two rudimentary ocellar median eyes), a third pair of ocellar eyes close to the larval brain, later merging with the probably chemosensory frontal organ, and numerous photo-sensors along the tail^114^), indicate that *Limulus* sp. represents an exception with numerous irregular and incomparable neoplasms. The conservative pycnogonids (Chelicerata) possess four ocellar median eyes, but in larval stages two of them each are innervated by a bifurcated nerve^51^, indicating that here a duplication or a splitting might have taken place—starting from two median eyes, and converging to four. This may have happened several times independently, or in one ancestor in common. The Cambrian “trilobitomorph” *C. eucalla* possessed four median eyes also, as did the leanchoiliids, and also phylogenetically old groups such as the crustacean phyllopods^115,116^ and some Collembola living today. Only pancrustaceans, (crustaceans and hexapods), are equipped with three median eyes. The number three probably arose by fusion of the median ocelli of the four. Accordingly, it seems that the plesiomorphic character for arthropods is two median eyes, as are still present in many Chelicerata. Four median eyes are apomorphic, perhaps even autapomorphic for *C. eucalla*, leanchoiliids and others, such as the chelicerate Pycnogonida, indicated by the bifurcate nerve, innervating two median eyes in the larval stages. Three median eyes are present in larval trilobites, and put them into the group of advanced euarthropods comparable to Pancrustacea, as this may be valid for the enigmatic five-eyed *Opabinia. regalis* Walcott, 1912 (Ecdysozoa, with uncertain systematic position), with presumably three median eyes and two main compound(?) eyes.

## Materials and methods

The photographs were taken with a Keyence digital-microscope (VHX-900F, VHZ-00R/0/T, VHZ-100R/W/T, VHZ-J20) at the Institute of Biology Education (Zoology), University of Cologne.

The specimens figured in this contribution.

*Aulacopleura koninckii* (Barrande, 1846), Na Černidlech Hill and Špičatý Hill near Loděnice in the Czech Republic (Silurian, Wenlock, Liten Formation, Motol Member, *Monograptus flexilis*-Zone); *Cindarella eucalla* Chen et al. 1996, Maotianshan Shales Quiongzhusi Section, Yu'anshan Member, Heilinpu Formation, Middle Early Cambrian, Mafang Village, Anning, Kunming, Yunnan Province, China; *Cyclopyge sibilla* Šnajdr, 1982. Ktaoua Fm., Upper Ordovician, El Kaid Errami, Morocco; *Eysyslopterus patteni* (Størmer, 1934), (Eurypterida), Silurian (Ludlow), Silma Cliff, Saaremaa, Estonia; *Eldredgeops (Phacops) rana crassituberculata* Stumm, 1953, Silica Shale Formation, Middle Devonian, Sylvania, Ohio, USA; *Gerastos cuvieri* (Steininger, 1831), Ahrdorf Fm., Flesten Mb., Middle Devonian, Gees/Gerolstein, Eifel, Germany; *Hermannina* sp., (Ostracoda), Silurian, Lickershamn, Gotland, Sweden; *Isotelus iowensis* Owen, 1852, Maquoketa Fm., Elgin Mt. Lower Ordovician, Rochmondian, Elgin, Iowa. *Leanchoilia superlata* Walcott, 1912, middle Cambrian, Burgess Shale, Canada; *Nyterops nyter* (Struve, 1970), Cürten Fm., Givetium, Middle Devonian, Weinsheim, Eifel, Germany.

## Supplementary Information

Supplementary Information.

## Data Availability

The material used during the current study are available from the corresponding author on reasonable request, and are deposited in collection of the Institute of Geology and Mineralogy of the University of Cologne.
