# Supplementary material for: The median eyes of trilobites
Source: Sci Rep. 2023 Mar 8;13:3917. doi: 10.1038/s41598-023-31089-7 (PMC9995485; doi:10.1038/s41598-023-31089-7)
Supplement: Supplementary file 1 — Supplementary Information. [file 41598_2023_31089_MOESM1_ESM.docx]

**S1 The enigmatic dorsal organs in trilobites – normally no median eyes**

For trilobites there has been a vigorous discussion from the end of the 19^th^ century and beginning of the 20^th^ century onwards focused on a small median tubercle, *the median sensory organ*. Such organs are present in several trilobite groups, but by no means all, and have been referred to under many different names (e.g. occipital organ^(1)^, Nackenschild, Dorsal organ^(2,3)^, DSO Dorsal sensory organ^(4)^, CMO Cephalic Median Organ^(5)^). This organ is positioned in the middle of the occipital ring (posteriormost of the fused segments of the cephalon). It forms a small dome in the cuticle, typically showing 5 pits, 4 arranged in a square and one slightly larger in the centre. Particularly fine examples are present in the silicified odontopleurids described by Whittington^(1)^, occuring in several genera. In *Diacanthaspis scitula* Whittington the occipital organ, both in the later meraspides growth stages and in the holaspides, is a pitted disc, borne on a short occipital spine, but many of the short glabellar spines each seem to terminate in an identical flat disc, with evident pits, perforated in a very similar manner. How to interpret these remains problematic. A similar, though less structurally defined occipital organ is found in *Olenus whalenbergi* Westergård, 1922^(6)^.

The *dorsal sensory organ*^(4)^ is situated in the centre, usually the highest point of the glabella. The best known examples are in the trinucleid *Tretaspis* sp*.*, elegantly described by Størmer^(7)^. Here there is median tubercle, with four pits arranged as on the corners of a square, around a larger central pit. This structure is arrestingly similar to the occipital organ of the odontopleurids, and it is most likely that they functioned in the same way, regardless of their position. Whether this is directly homologous with the occipital organ as in odontopleurids, though shifted forwards, remains an open question. Lerosey-Aubril & McNamara^(5)^, consider that it is, as in many asaphids. The asaphid *Nileus sp.* has a different kind of dorsal org an; a thin patch in the centre of the glabella, which Fortey & Clarkson^(8)^, studied in detail, and on the basis of thin sections, envisaged as light-sensitive.

The dorsal tubercle has been interpreted as beginning of the alimentary canal^(9)^, a dorsal organ^(10)^, or especially in trinucleid trilobites as a simple eye^(8,11,12)^. Ruedemann^(13)^, was one of the first to consider these as “median eyes”. He gives a review of where to find these structures among trilobites, and describes, on the basis of thin sections of *Cryptolithus* sp. and *Asaphus* sp. a thin shell above the ocellus, and a small lenticular body below the shell. Richter^(12, p.105)^ interprets the same as a late filling of an empty cavity. Ruedemann also noted some carbonaceous relics on the underside of the ocellus, and interprets it as traces of a small retina^(13, p.235)^. Størmer^(7)^, followed on by describing the fine-structure of the tubercular organ. He found in *Tretaspis* sp. that the internal structure of the four outer pits was different from that of the central pit. He interpreted the outer structures as retinas of median eyes, the inner as relic of an eye sac. Hanström^(3)^, takes up these results and emphasises the similarity of this organ to the dorsal organ of crustaceans, to which trilobites at that time were systematically ascribed. At the back of the head of *Anaspides tasmaniae* (Thomson, 1893), (Anaspidacea, Malacostraca), as present also in other Malacostraca such as Phyllopds and Syncarida, he finds an organ, which he calls “four-celled-organ” strikingly similar in its structure to those of the trilobite *Tretaspis* sp.. His detailed morphological analysis reveals that the four outer systems contain one bipolar sensory cell each, and the central one, characterised by its thinned cuticle, is set in connection to a larval respiratory organ of phyllopods^(14)^. The “four-celled-sensory organ” is connected by a nerve with a tritocerebrum, a fact which we shall take up later on.

It is not until 74 years later that the cephalic median organ of trilobites returned to be a focus of interest. Lerosey and McNamara^(5)^, describe the distribution of these rare features in trilobites, which occur from the middle Cambrian onwards, but because of the great constancy of the organ in terms of morphology and of positioning they suggest that it might have been inherited from a common ancestor of all trilobites. They point out that for example in *Aulacopleura koninckii* var. *occitanica* Chaubet, 1937 the organ (Fig. 1d,e) can be found only in the internal moulds, but cannot be seen from the outside in intact specimens. This may be a reason why this organ has remained generally unnoticed. From outside it can be clearly observed in *Isotelus sp.* (Fig. 1a). Since the dorsal organ does not occur in proetids it disappears after the Devonian. There can be, however, be drawn a parallel between the absence of this central median organ (CMO) and the existence of an occipital node in this group^(4,8,15)^. This may be similar to that of certain Devonian phacopid trilobites (Fig. 1b,c), which show no CMO, but have a distinct node on the occipital ring. In 2013 Lerosey and Meyer^(16)^ analysed the CMO, here called SDO (sensory dorsal organ) in larvae and adult of various malacostracan crustaceans. The SDO is composed of four chemo-sensors, and a larger gland in the centre, positioned on a thinned cuticle. The gland consists of a single cell, and it is suggested that the chemo-sensors are functionally associated with the gland. Its occurrence in non-crustacean Cambrian arthropods (Orsten) indicates that these organs already existed in the early history of crustaceans and is essential to their functioning and it probably was essential for trilobites also. In malacostracans there is sometimes a second organ very similar to the SDO positioned more posteriorly on the carapace; this is the posterior SDO. It is very similar to the SDO, but has a greater number of sensors (usually six to ten) ^(16)^, and thus very much resembles the more complex CMOs of *Olenus whalenbergi* Westergård, 1922^(6)^.

The function of dorsal organs had been discussed frequently^(e.g. 16-19)^, and it is highly probable that the complex of 4 chemosensory cells and a central presumed salt-gland^(20)^, functions in larval crustaceans and, as we think, in trilobites as an organ which stabilises homoeostasis, especially in larvae living in shallow waters close to the shores, with often and quickly changing osmotic conditions. The dorsal organ degenerates in many crustaceans during ontogeny^(19,20)^, because the gills take over the osmoregulatory function. For the same reason DROs may disappear in many adult forms of trilobites.

Although it is likely that the term “dorsal organ” is used for a variety of cuticular organs^(17)^, the similarity in the arrangement of its elements and their fine-structure (four minute pits around a central pore forming a quincunx, and a thinned cuticle below the pits, encircled by a cuticular border) suggest that the trilobite-DMOs, at least some of them (see below), are indeed chemoreceptors combined with a gland. The fact that Lerosey and Meyer 2013^(16)^, found SDO/DMO-like organs in non-crustacean arthropods from the Late Cambrian Alum Shale of Sweden, strengthens this idea and strongly suggests that the origin of this organ lies deeply in the early history of arthropods. The fact, that the SDO is innervated by the tritocerebrum^(2, p.98, 17)^, however, makes clear that the SDO/DMO is not homologous with the protocerebrally innervated median eyes.

There is an interesting note, however, made by Ruedemann in 1916 about *Cryptolithus (Trinucleus) tesselatus* Green, 1832 from the Trenton Limestone, New York. When the presumed cornea of the single glabellar tubercle was removed, he found, on the underside, a black carbonaceous layer. He interpreted this structure in modern words as an ocellus, consisting of a lens- or pear-shaped sac, covered by a thin cuticular membrane and filled with fluid, functioning as a lens, comparable to the Nauplius (parietal) eyes of other crustaceans, namely phyllopods^(13, p. 136)^. The carbonaceous layer is seen as a relic of the pigmented retina.

A similar report is given by Hanström^(2,3)^ about *Aeglina prisca* (Barrande, 1872), (later *Pricyclopyge prisca).* *P. prisca* is a pelagic trilobite with conspicuously large compound eyes, adapted for swimming upside down. On its glabella there are three smaller structures, which Hanström interprests as relics of a highly developed divided Nauplius eye, comparable to that of coryceid and pontellid (both copepod) crustaceans. If this was the case it seems to be very likely that the glabellar tubercles^(21)^, may actually be (at least) two different structures – a chemosensory DMO or indeed median eyes.


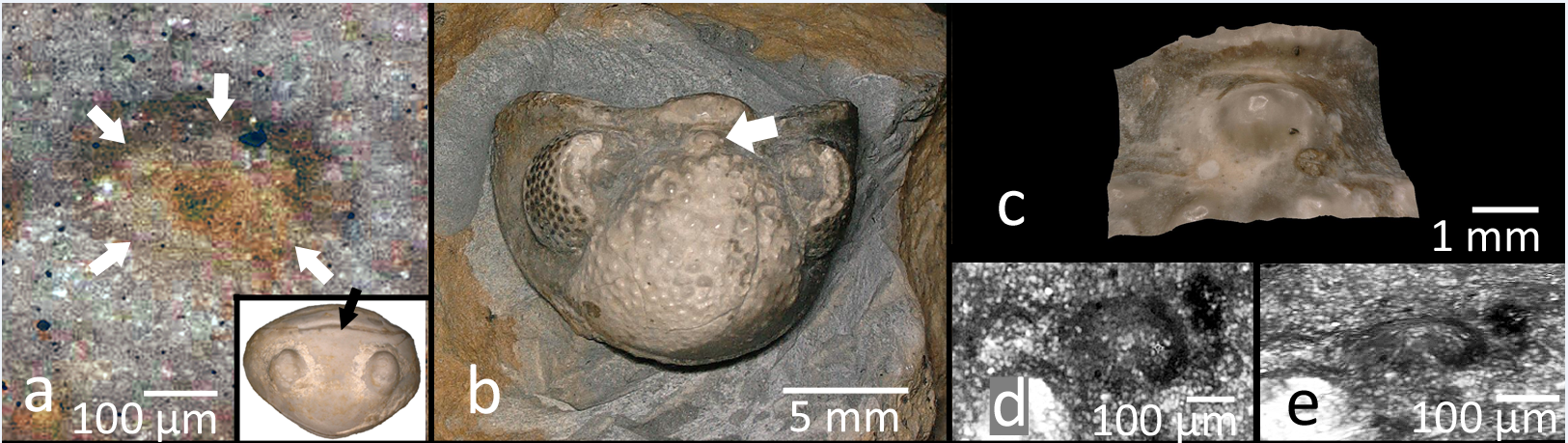


**Figure 1 Dorsal organs of two trilobites, and the glabellar node of *Nyterops nyter* (Struve, 1970**)

a) Dorsal organ of *Isotelus iowensis* Owen, 1852, Ordovician, USA (insert). Arrows indicate presumed chemosensoric structures, assumed salt gland in the center. b) Glabellar node (white arrow) of *Nyterops nyter* (Struve, 1970), Middle Devonian, Germany. c) Enlargement of glabellar node of b). d) dorsal organ of *Cyclopyge sibilla* Šnajdr, 1982 (yellow arrow in Fig. 1l). e) lateral view of d).

References

1. Whittington, H. B. Type and other species of Odontopleuridae (Trilobita). *J. Paleontol.* **30**, 504-520 (1956).

2. Hanström, B. Neue Untersuchungen über Sinnesorgane und Nervensystem der Crustaceen. I. *Z.* *Morph. Oekol. Tiere* **23***,* 80–236 (1931).

3. Hanström, B. Ueber das Vorkommen eines Nackenschildes und eines vierzelligen Sinnesorganes bei den Trilobiten. *Lunds Univ. Arskr., N. F.* **2***,* (1934).

4. Lerosey-Aubril, R., McNamara, K. J., Rábano, I., Gozalo, R. & García-Bellido, D. The cephalic median organ of trilobites in *Advances in trilobite research* (eds. Rábano, I. & Gozalo, R.) 229-235 (Instituto Geológico y Minero de España, Madrid, 2008).

5. Lerosey‐Aubril, R. & Meyer, R. The sensory dorsal organs of crustaceans. *Biol. Rev.* **88**, 406-426 (2013).

6. Clarkson, E. N. K. & Taylor, C. M. (1995). Ontogeny of the trilobite *Olenus wahlenbergi* Westergård, 1922 from the upper Cambrian Alum Shales of Andrarum, Skåne, Sweden. *R. Soc. Edinb. Earth Sci.* **86**, 13-34.

7. Størmer, L. Scandinavian *Trinucleidae*, with special references to Norwegian species and varieties*. Skrift. Norske Vidensk. Acad.* **5**, 1-111 (1930).

8. Fortey, R. A. & Clarkson, E. N. K. The function of the glabellar ‘tubercle’ in Nileus and other trilobites. *Lethaia* **9**, 101-106 (1976).

9. Beyrich, E. *Ueber einige böhmische Trilobiten: zweites Stück*. (Reimer, Berlin, 1846).

10. Bernard, H. M. The systematic position of the trilobites. *Quart. J. Geol. Soc.* **50**, 411-434 (1894).

11. Raymond, P. E. Phylogeny of the *Arthropoda* with especial reference to the trilobites. *Am. Nat.* **54**, 398-413 (1920).

12. Richter, R. Beiträge zur Kenntnis devonischer Trilobiten. *Ab. Senckenb. Naturf. Gesell.* **37**, 177-218 (1921).

13. Ruedemann, R. On the presence of a median eye in trilobites. *PNAS* **2**, 234-237 (1916).

14. Dejdar, E. Die Korrelationen zwischen Kiemensäckchen und Nackenschild bei Phyllopoden. *Z. Zool.* **136**, 422-432 (1930).

15. Lerosey-Aubril, R. & Feist, R. First carboniferous protaspid larvae (Trilobita). *J. Paleont.* **79**, 702-718 (2005).

16. Barrientos, Y. & Laverack, M. S. The larval crustacean dorsal organ and its relationship to the trilobite median tubercle. *Lethaia* **19**, 309-313 (1986).

17. Martin, J. W. & Laverack, M. S. On the distribution of the crustacean dorsal organ. *Acta Zool.* **73**, 357-368 (1992).

18. Roberston, S. The dorsal organ: what is it and what is it for? *The Plymouth Student Scientist* **6**, 412-433 (2013).

19. Thabet, R., Ayadi, H., Koken, M. & Leignel, V. Homeostatic responses of crustaceans to salinity changes. *Hydrobiologia* **799**, 1-20 (2017).

20. Charmantier, G. U. Y. Ontogeny of osmoregulation in crustaceans: a review. *Inv. Repr. Dev.* **33***,* 177-190 (1998).

21. McCormick, T. & Fortey, R. A. Independent testing of a paleobiological hypothesis: the optical design of two Ordovician pelagic trilobites reveals their relative paleobathymetry. *Paleobiology* **24**, 235-253 (1998).
